# Heart Failure with Reduced versus Preserved Ejection Fraction: Molecular Mechanisms, Immunologic Pathways, Current Therapies, and Future Directions

**DOI:** 10.26502/aimr.0254

**Published:** 2026-08-27

**Authors:** Erin Kim, Devendra K. Agrawal

**Affiliations:** 1 Western University College of Osteopathic Medicine of the Pacific, Pomona, CA, USA.; 2 Department of Translational Research, College of Osteopathic Medicine of the Pacific, Western University of Health Sciences, Pomona, CA, USA.

**Keywords:** Cardiomyocyte dysfunction, Coronary microvascular disease, Endothelial dysfunction, Fibrosis, Guideline-directed medical therapy, Heart failure, HFrEF, HFpEF, Inflammation, Mitochondrial dysfunction, Molecular phenotyping, Social determinants of health

## Abstract

Heart failure remains a major clinical and public health challenge due to its rising prevalence, heterogeneous biology, and persistent burden of morbidity, mortality, and healthcare utilization. Although left ventricular ejection fraction remains central to classification and treatment selection, it incompletely captures the molecular and clinical diversity of heart failure syndromes. Heart failure with reduced ejection fraction is commonly characterized by cardiomyocyte injury, impaired systolic force generation, neurohormonal activation, and adverse ventricular remodeling, which has enabled development of a standardized guideline-directed medical therapy framework. In contrast, heart failure with preserved ejection fraction is driven by more heterogeneous and overlapping mechanisms, including cardiometabolic stress, endothelial dysfunction, coronary microvascular disease, inflammation, myocardial fibrosis, and impaired physiologic reserve. This narrative review synthesizes current evidence on the fundamental pathophysiology, cellular and molecular mechanisms, immunologic pathways, and contemporary therapeutic strategies in HFrEF and HFpEF. Emphasis is placed on the translational gap between mechanistic discovery and clinically actionable treatment selection. Emerging evidence suggests that future progress, especially in HFpEF, may depend on integrating molecular phenotyping with biomarkers, imaging, hemodynamics, comorbidity profiles, and social determinants of health. A more precise and equitable approach to heart failure care may improve risk stratification, therapeutic selection, and patient-centered outcomes across the full spectrum of disease.

## 1. Introduction

Heart failure (HF) represents a growing clinical and public health challenge, driven by population aging, improved survival from cardiovascular disease, and the increasing burden of cardiometabolic comorbidities [1]. Although HF has historically been viewed as a disease of older adults, contemporary trends suggest that its burden is extending into younger and middle-aged populations, likely reflecting earlier accumulation of risk factors such as obesity, hypertension, diabetes, and chronic kidney disease [1]. Indeed, the concurrent heart failure, atrial fibrillation/flutter, and sepsis significantly increase the mortality [2].

The epidemiology of HF is heterogeneous and varies meaningfully across demographic and geographic groups. Age remains one of the strongest determinants of HF prevalence, but sex, race, ethnicity, and social context also shape disease distribution. Black individuals experience a disproportionately high burden of HF, including higher incidence and prevalence compared with other racial and ethnic groups. At the same time, increasing prevalence has also been observed among Hispanic/Latino populations [1]. These differences are unlikely to be explained by biological risk alone and instead reflect the cumulative influence of cardiometabolic risk-factor clustering, structural inequities, social determinants of health, environmental exposures, and differences in access to preventive and longitudinal cardiovascular care.

The impact of risk factors is central to HF development and progression. Hypertension and coronary artery disease are major contributors, while obesity, diabetes mellitus, smoking, atrial fibrillation, and chronic kidney disease further increase the risk [1]. Importantly, these risk factors do not contribute uniformly across HF phenotypes. HF with preserved ejection fraction (HFpEF) is especially associated with obesity, hypertension, metabolic syndrome, diabetes, and renal dysfunction [3]. HF with reduced ejection fraction (HFrEF) is more often linked to myocardial injury, ischemic heart disease, and adverse ventricular remodeling, although hypertension remains a major contributor as well [1]. In the United States, HF has a disease prevalence of around 6.7 million people. HFpEF accounts for more than half of these patients [2,3,4]. The overall prevalence of HF is projected to rise substantially over the coming decades, underscoring its expanding contribution to morbidity, mortality, healthcare utilization, and economic burden.

Together, these epidemiologic trends highlight the need to conceptualize HF not as a single disease, but as a heterogeneous clinical syndrome arising from diverse structural, metabolic, neurohormonal, and immune-mediated pathways. This distinction is particularly important when comparing HFrEF and HFpEF. While HFrEF has a well-established foundation of guideline-directed medical therapy, HFpEF remains more difficult to treat. HFpEF also remains underrecognized in clinical practice, in part because its varied clinical presentation often creates greater diagnostic uncertainty [5]. Accordingly, this review summarizes the fundamental pathophysiology of HF, compares the cellular and immunologic mechanisms underlying HFrEF and HFpEF, reviews current therapeutic strategies, and identifies key knowledge gaps that may guide future research and clinical care.

## 2. Definitions and Classification of Heart Failure

HF is a clinical syndrome that develops when structural or functional cardiac abnormalities impair ventricular filling, ventricular ejection, or both. The diagnosis is made with a combination of clinical symptoms such as dyspnea, fatigue, exercise intolerance, and edema, with objective evidence of cardiac dysfunction.

HF is commonly categorized using both stage-based and left ventricular ejection fraction (LVEF)-based systems. The ACC/AHA staging framework emphasizes HF as a progressive disease continuum. Stage A includes patients at risk for HF, such as those with hypertension, diabetes, obesity, atherosclerotic cardiovascular disease, cardiotoxic exposures, or genetic susceptibility, but without symptoms or structural heart disease. Stage B describes asymptomatic patients with evidence of structural heart disease, abnormal cardiac function, elevated filling pressures, or abnormal cardiac biomarkers. Stage C includes patients with current or prior symptoms of HF in the setting of structural heart disease, while Stage D refers to advanced HF with persistent symptoms, recurrent hospitalizations, or need for specialized therapies despite attempts to optimize guideline-directed medical therapy [6].

LVEF-based classification remains central to prognosis, clinical trial design, and treatment selection. HFrEF is defined by an LVEF of 40% or less, HFmrEF by an LVEF of 41% to 49%, and HFpEF by an LVEF of 50% or greater. For HFmrEF and HFpEF, the presence of preserved or mildly reduced systolic function alone is insufficient; diagnosis also requires evidence of increased filling pressures through natriuretic peptides, cardiac imaging, or invasive hemodynamic assessment [6].

Although these classification systems are clinically useful, they do not fully reflect the biological complexity of HF. Patients within the same LVEF category may differ substantially in etiology, comorbidity burden, ventricular remodeling, inflammatory signaling, and response to therapy. This limitation is particularly relevant to HFpEF, which often arises from multiple overlapping cardiovascular and systemic processes. Therefore, staging and LVEF-based classification provide an essential clinical framework, but a deeper mechanistic understanding is necessary to explain the distinct pathophysiology and treatment responsiveness of HFrEF and HFpEF.

## 3. Fundamental Pathophysiology of HFpEF and HFrEF

**Figure 1:**
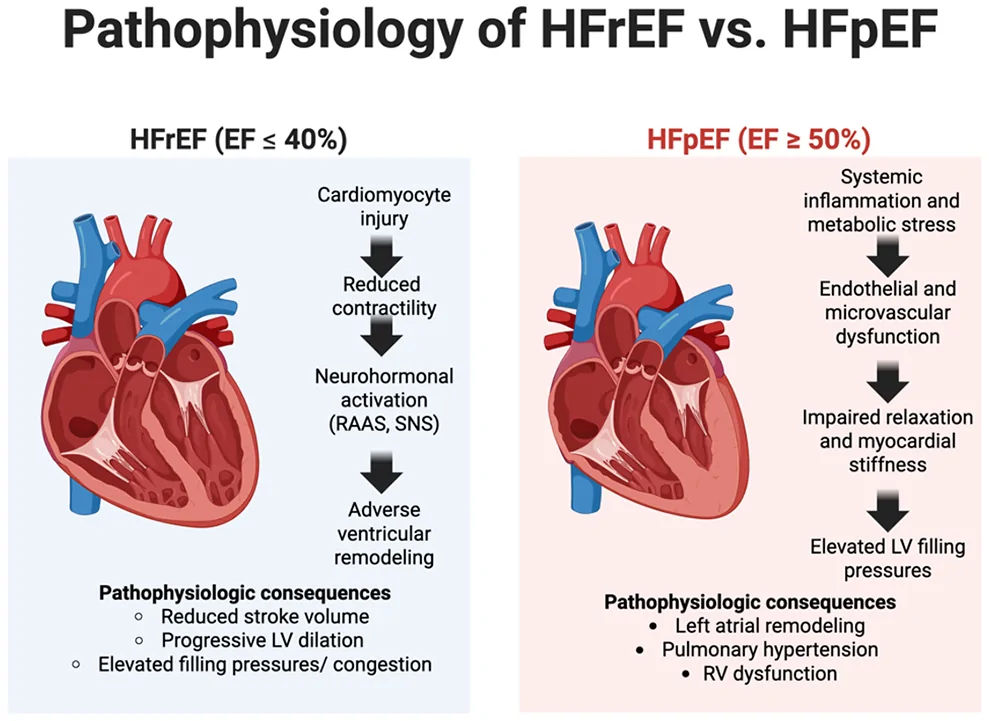
Pathophysiology of HFrEF and HFpEF. This figure shows the key features of the dominant pathophysiologic mechanisms and downstream consequences of heart failure with reduced and preserved ejection fraction.

The key features of the dominant pathophysiologic mechanisms and downstream consequences of heart failure with reduced and preserved ejection fraction are schematically shown in Figure 1.

HFpEF is a clinical syndrome in which patients develop symptoms and signs of heart failure despite having a left ventricular ejection fraction that is normal or near normal [4,7,8]. Preserved ejection fraction does not imply normal cardiac function. Rather, HFpEF is commonly characterized by impaired ventricular relaxation, increased myocardial stiffness, abnormal vascular function, and a high burden of systemic comorbidities [7-9]. A central physiologic feature is an abnormal rise in left ventricular filling pressures, which may be present at rest or become evident only during exertion. This increase in filling pressure contributes to dyspnea, pulmonary congestion, reduced exercise capacity, and functional limitation [7,8].

Early models of HFpEF emphasized hypertension-related left ventricular hypertrophy and diastolic dysfunction. In this framework, chronic pressure overload produces a smaller and less compliant ventricle, which impairs relaxation and raises filling pressures [7]. Although this mechanism remains important, HFpEF is now understood as a broader syndrome shaped by systemic comorbidity burden. Conditions such as obesity, diabetes, chronic kidney disease, atrial fibrillation, and physical inactivity can create a chronic inflammatory and metabolic environment that affects both the myocardium and the peripheral circulation [4,7,8]. Over time, this systemic stress can impair endothelial function and limit myocardial reserve, allowing heart failure symptoms to develop even when systolic contractility is relatively preserved [5,7,9] (Figure 1).

HFpEF also extends beyond isolated left ventricular dysfunction. Persistently elevated left-sided filling pressures expose the left atrium to chronic pressure stress, which can promote atrial remodeling and increase susceptibility to atrial fibrillation [7]. As this pressure burden progresses, it may extend into the pulmonary circulation and contribute to pulmonary hypertension and right ventricular dysfunction [7,8]. Functional limitation in HFpEF is also influenced by abnormalities outside the left ventricle. Some patients develop poor heart rate augmentation during exertion, while others have impaired vascular reserve, skeletal muscle dysfunction, or comorbid pulmonary and renal disease that further limits exercise capacity [5,7,8]. As a result, the clinical presentation of HFpEF reflects the combined effects of myocardial stiffness, vascular dysfunction, pulmonary pressure overload, metabolic stress, and peripheral limitations. The relative contribution of each mechanism differs across patients, so HFpEF symptoms often reflect more than diastolic dysfunction alone [7,8]. This complexity underscores the need to evaluate HFpEF as a multisystem syndrome shaped by cardiac dysfunction, vascular abnormalities, extracardiac comorbidities, and limitations in physiologic reserve [5,7,8].

HFrEF develops when the left ventricle can no longer generate enough systolic force to maintain effective forward circulation. It is typically defined by a left ventricular ejection fraction of 40% or less. It is also commonly associated with progressive ventricular dilation and adverse remodeling [10]. The initial insult may vary. In some patients, HFrEF follows ischemic myocardial injury, while in others it develops from chronic pressure or volume stress, inherited cardiomyopathy, myocarditis, persistent tachyarrhythmia, or cardiotoxic exposure. Despite these different causes, many cases converge on a shared pathway in which cardiomyocyte injury weakens contractility, lowers stroke volume, and activates compensatory neurohormonal systems [10,11] (Figure 1).

The sympathetic nervous system and renin-angiotensin-aldosterone system are initially activated to preserve perfusion. By increasing heart rate, contractility, vascular tone, and intravascular volume, these responses can temporarily support cardiac output [11]. Chronic sympathetic and RAAS signaling increases myocardial oxygen demand, promotes sodium and water retention, and raises ventricular loading conditions. These changes place additional stress on an already weakened ventricle and stimulate pathologic remodeling through cardiomyocyte hypertrophy, cell death, fibroblast activation, and interstitial fibrosis [11]. Over time, these processes worsen ventricular structure and function, creating a self-perpetuating cycle of worsening ventricular dysfunction. The left ventricle becomes progressively dilated, less efficient, and less able to generate forward stroke volume. As systolic function declines, patients develop reduced cardiac reserve, elevated filling pressures, congestion, and exercise intolerance [9,10]. Thus, HFrEF is not simply a fixed reduction in ejection fraction. It is a progressive syndrome in which myocardial injury, compensatory neurohormonal activation, and structural remodeling reinforce one another over time.

Although HFpEF and HFrEF differ in their dominant hemodynamic patterns, both arise from coordinated changes across multiple cardiac and extracardiac cell types. The following section examines the cellular and molecular mechanisms that drive these phenotypes, with emphasis on cardiomyocytes, fibroblasts, endothelial cells, and inflammatory cell populations.

## 4. Cellular and Molecular Mechanisms in HFrEF and HFpEF

**Figure 2:**
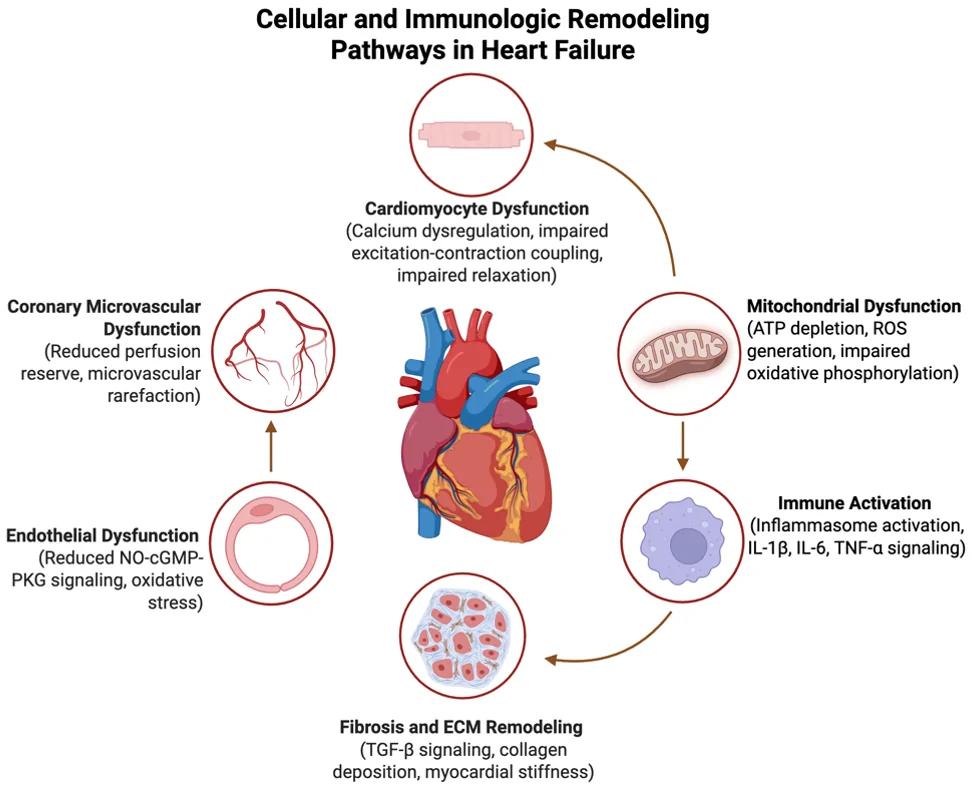
Cellular and immunologic remodeling pathways in heart failure. These include cardiomyocyte dysfunction, mitochondrial dysfunction, immune activation, endothelial dysfunction, coronary microvascular dysfunction, and fibrosis with extracellular matrix remodeling.

The cellular and immunological pathways in the heart failure are shown in Figure 2 and discussed in the following sections.

### 4.1 Cardiomyocyte Dysfunction

Cardiomyocytes are central to the development of both HFrEF and HFpEF, but the nature of their dysfunction differs by phenotype. In HFrEF, the primary abnormality is failure of the myocyte to generate adequate systolic force. This reflects disruption of the contractile apparatus and the cellular systems that coordinate contraction. Sarcomere function becomes less efficient, excitation-contraction coupling becomes less synchronized, and calcium cycling becomes impaired. Together, these changes weaken contraction, reduce contractile reserve, and contribute to progressive loss of myocardial performance [12-14].

Abnormal calcium handling is a key mechanism of cardiomyocyte dysfunction in heart failure. Disruption of transverse tubules can impair electrical signaling and alter sodium and calcium balance, weakening the coordination between membrane depolarization and myofilament contraction [12]. In sarcomere disease, increased calcium sensitivity may produce hypercontractility during systole while impairing relaxation during diastole, which increases ATP demand and reduces mechanical efficiency [12,13]. Normally, calcium entry through L-type calcium channels triggers calcium release from the sarcoplasmic reticulum through ryanodine receptors, allowing myofilament contraction. Relaxation then depends mainly on SERCA-mediated calcium reuptake into the sarcoplasmic reticulum, with additional calcium extrusion through the sodium-calcium exchanger [15] (Figure 2).

In heart failure, this system becomes disrupted. In HFrEF, reduced SERCA function, altered phospholamban regulation, increased sodium-calcium exchanger activity, and ryanodine receptor calcium leak reduce sarcoplasmic reticulum calcium stores and weaken the systolic calcium signal [15]. These changes impair contraction and delay relaxation, while diastolic calcium leak may increase arrhythmia risk. Remodeling of transverse tubules further disrupts excitation-contraction coupling by weakening the normal alignment between L-type calcium channels and ryanodine receptors [15]. In a nonischemic experimental HF model, RyR2 hyperactivity promoted diastolic calcium leak with reduced sarcoplasmic reticulum calcium stores and impaired systolic contraction, while RyR2 inhibition reduced arrhythmic death and improved cardiac performance [16]. Therefore, altered calcium cycling in HFrEF links cellular dysfunction to impaired contractility, delayed relaxation, and reduced myocardial reserve.

In HFpEF, calcium-handling abnormalities are less clearly defined than in HFrEF, but they appear to affect relaxation more than systolic contraction. Some experimental models suggest that calcium transients may be preserved or even increased early in disease [15]. However, if calcium reuptake is delayed or diastolic calcium remains elevated, cardiomyocytes may relax more slowly. This becomes especially important during exercise, when faster heart rates shorten diastolic filling time and increase the demand for rapid relaxation. In this setting, impaired calcium cycling may contribute to elevated filling pressures and reduced exercise capacity despite preserved ejection fraction [15].

Cardiomyocyte dysfunction in HFpEF is therefore less defined by loss of systolic force and more by increased stiffness and limited reserve during physiologic stress. Titin is central to this process because it functions as the major elastic protein within the sarcomere and helps determine passive cardiomyocyte stiffness [14]. When titin isoform expression, phosphorylation, or cleavage is altered, the cardiomyocyte becomes less compliant and ventricular filling becomes more impaired [13,14]. In HFpEF, reduced nitric oxide-cGMP-PKG signaling and increased PKC activity have been linked to titin-based stiffening, providing a molecular connection between systemic cardiometabolic stress and impaired myocardial compliance [14].

These differences highlight the distinct cellular basis of HFrEF and HFpEF. In HFrEF, cardiomyocyte dysfunction is driven primarily by impaired systolic force generation and loss of contractile reserve. In HFpEF, the abnormality is more closely related to delayed relaxation, increased passive stiffness, and limited adaptability during exertion. Despite these differences, both phenotypes are shaped by disturbances in calcium handling, sarcomere function, and titin regulation, which contribute to the transition from compensated myocardial stress to symptomatic heart failure [12-14,17].

### 4.2 Mitochondrial Dysfunction, Oxidative Stress, and Metabolic Remodeling

Mitochondrial dysfunction is a central contributor to heart failure because cardiomyocytes require continuous ATP production to support contraction, relaxation, ion transport, and calcium reuptake. In the healthy adult heart, mitochondrial oxidative metabolism allows energy production to match mechanical demand. In heart failure, this balance is lost. Neurohormonal activation, pathologic remodeling, and impaired calcium handling increase energetic demand, while mitochondrial dysfunction reduces the efficiency of ATP generation [18,19,20]. This energetic mismatch limits contractile reserve and contributes to exercise intolerance, impaired relaxation, and progression from compensated stress to symptomatic heart failure.

Metabolic remodeling further reduces cardiac efficiency. The adult heart normally relies heavily on fatty acid oxidation, but the failing heart often shifts toward greater glucose use and glycolysis [18,19]. Although this change may initially help preserve ATP production during stress, glycolysis is less efficient than oxidative phosphorylation and may become uncoupled from glucose oxidation, reducing overall ATP efficiency (18). Impaired fatty acid oxidation can also promote lipid accumulation and metabolic stress, leaving the myocardium less flexible in how it generates energy [18,19]. As a result, the failing heart becomes increasingly dependent on inefficient pathways that are less able to support increased physiologic demand.

Mitochondrial dysfunction also promotes oxidative injury. Excess reactive oxygen species can damage mitochondrial proteins, lipids, and DNA, which further impairs respiratory chain function and amplifies cellular stress [18,19,20]. Calcium overload and oxidative stress may also trigger opening of the mitochondrial permeability transition pore, leading to loss of membrane potential, ATP depletion, mitochondrial swelling, and activation of cell death pathways [18,19,20]. Thus, mitochondria contribute to heart failure not only by producing less energy, but also by promoting oxidative damage and maladaptive remodeling (Figure 2).

Mitochondria-associated endoplasmic reticulum membranes (MAMs) provide an additional link between calcium handling and energy metabolism. MAMs help coordinate calcium transfer from the endoplasmic reticulum to mitochondria, allowing ATP production to rise with contractile demand [19]. In heart failure, disruption of this communication can worsen calcium overload, reduce oxidative capacity, and further impair energetic reserve. Across both HFrEF and HFpEF, the shared consequence is the reduced ability of the myocardium to increase performance during physiologic stress. Therefore, mitochondrial dysfunction, oxidative stress, and metabolic remodeling represent a common pathway linking cellular injury to exercise intolerance and progressive heart failure [19,20].

### 4.3 Fibroblast Activation and Extracellular Matrix Remodeling

Fibroblast activation and extracellular matrix (ECM) remodeling are central mechanisms in heart failure progression. In healthy myocardium, cardiac fibroblasts help preserve tissue structure. When the heart is exposed to injury or sustained mechanical, metabolic, or inflammatory stress, these cells can shift toward an activated myofibroblast phenotype [21,22]. Activated fibroblasts proliferate, migrate, and increase production of ECM proteins, particularly collagens I and III, fibronectin, and proteoglycans. Over time, the accumulating ECM stiffens the myocardium, impairs ventricular relaxation, disrupts electrical conduction, and contributes to progressive cardiac dysfunction [21,22].

The pattern of fibrosis differs across heart failure phenotypes. In HFrEF, particularly after myocardial infarction or ischemic injury, fibrosis often develops as replacement scar in areas of cardiomyocyte loss. This scar is important for preserving structural integrity after injury, but it also contributes to adverse remodeling, reduced contractile reserve, and impaired ventricular mechanics [21,23]. In HFpEF, fibrosis is more often diffuse and interstitial. Rather than replacing a focal area of necrosis, the matrix expands gradually in response to chronic pressure stress and systemic cardiometabolic disease [21,22]. This pattern increases myocardial stiffness and contributes directly to elevated filling pressures and impaired diastolic function.

ECM remodeling depends not only on the amount of collagen deposited, but also on how the matrix is organized and turned over. Collagen cross-linking, collagen subtype balance, and matrix degradation all influence myocardial stiffness and reversibility. Collagen I tends to create a more rigid matrix, whereas collagen III contributes more to tissue elasticity [24]. Matrix metalloproteinases and their inhibitors regulate ECM degradation and turnover, while profibrotic mediators through pathways such as TGF-β promote fibroblast activation, collagen deposition, and matrix stabilization. As the ECM becomes stiffer, mechanical feedback can sustain myofibroblast differentiation, creating a self-reinforcing cycle of fibrosis and impaired myocardial compliance [22].

Fibrosis can represent both an adaptive repair response and a maladaptive driver of heart failure. After acute injury, fibroblast activation helps stabilize damaged myocardium. However, persistent activation promotes ECM accumulation leading to ventricular stiffening and impaired myocardial function. Clinical biomarker studies support this concept, as lower levels of procollagen type I C-terminal propeptide have been associated with greater left ventricular reverse remodeling and fewer HF-related outcomes in patients with HF and LVEF below 50% [25]. Therefore, fibroblast activation and ECM remodeling provide an important cellular link between myocardial stress, inflammation, and progressive heart failure [21-24].

### 4.4 Endothelial Dysfunction and Coronary Microvascular Disease

Endothelial dysfunction and coronary microvascular disease provide an important vascular mechanism for HFpEF. Under normal conditions, the coronary microcirculation increases myocardial blood flow when metabolic demand rises. When this reserve is impaired, the myocardium becomes more vulnerable to ischemic and energetic stress during exertion, even without obstructive epicardial coronary artery disease [26-28]. Clinically, this impairment is often reflected by reduced coronary flow reserve, which has been increasingly recognized as a marker of microvascular dysfunction in HFpEF [26,27]. In an invasive coronary physiology study of HFpEF patients without significant epicardial coronary stenosis, coronary microvascular dysfunction (CMD) was present in most patients with both endothelium-dependent and endothelium-independent abnormalities [29]. This finding supports CMD as a common but biologically heterogeneous feature of HFpEF rather than a single vascular abnormality.

In HFpEF, systemic comorbidities can promote microvascular injury through chronic inflammation and oxidative stress. This inflammatory environment reduces endothelial nitric oxide bioavailability and weakens NO-cGMP-PKG signaling, a pathway that normally supports vasodilation and myocardial compliance [26,30,31]. As this signaling declines, the ventricle becomes less able to relax and accommodate increased filling demands. This mechanism is especially relevant during exercise, when impaired vasodilatory reserve and reduced myocardial compliance can raise filling pressures and worsen symptoms.

Structural changes within the microcirculation may further reinforce this process. Microvascular rarefaction reduces the density of small vessels available to deliver oxygen to the myocardium, while abnormal microvascular remodeling can limit perfusion reserve during stress [26,30,31]. Over time, impaired perfusion may promote cardiomyocyte stress and fibrotic remodeling, while the remodeled myocardium can further compromise microvascular function [26,30,32]. This bidirectional relationship helps explain how HFpEF can progress despite relatively preserved systolic contractility. In the invasive HFpEF study, impaired endothelium-independent microvascular function was associated with lower diastolic relaxation velocity, higher estimated filling pressures, and increased mortality, further linking CMD to diastolic dysfunction and adverse outcomes [29].

Microvascular dysfunction is not exclusive to HFpEF. In HFrEF, endothelial injury and impaired microvascular perfusion can worsen oxidative stress, neurohormonal activation, and adverse ventricular remodeling [28]. However, CMD appears to be particularly central in HFpEF because it connects systemic comorbidity burden with myocardial stiffness and diastolic dysfunction.

### 4.5 Genetic Architecture and Molecular Susceptibility

Genetic susceptibility adds an important dimension to the molecular complexity of heart failure. In a large genome-wide association study of HF and its subtypes, multiple loci were associated with overall HF, nonischemic HF, and EF-based subgroups, supporting the idea that inherited risk contributes to HF biology beyond traditional acquired risk factors [33]. These findings suggest that HF does not arise from a single genetic pathway. Instead, genetic risk appears to be distributed across processes that influence myocardial structure, contractile function, vascular biology, metabolism, and extracardiac organ systems

The genetic architecture also appears to differ between HFrEF and HFpEF. Nonischemic HFrEF demonstrated stronger SNP-based heritability than nonischemic HFpEF [33]. In this group of nonischemic HFrEF, genes were enriched in pathways related to cardiac development, sarcomeric function, hypertrophy, dilated cardiomyopathy, and arrhythmia [30]. These findings align with the clinical pattern of HFrEF, in which impaired contractile function often reflects direct myocardial injury or structural cardiomyocyte disease.

In contrast, the genetic signal for HFpEF appears to reflect a broader systemic biology. The genes in the HFpEF group showed enrichment for kidney and pancreatic tissues, along with stronger links to body mass index. This supports the concept that HFpEF is often shaped by cardiometabolic and renal pathways rather than by a purely myocardial process [33]. Genetic analyses also suggested phenotype-specific relationships between major risk factors, with systolic blood pressure showing a stronger association with nonischemic HFrEF and body mass index showing a larger effect on nonischemic HFpEF [33]. This distinction reinforces the idea that preserved EF does not signify a single disease mechanism.

Transcriptomic and bioinformatics approaches provide a complementary view of HF biology. One study that integrated differential gene expression, co-expression network analysis, and machine-learning methods identified HMGN2, HTRA1, MFAP4, and MYH6 as candidate molecular markers associated with HF [34]. These genes map onto several core pathways discussed in this review, including sarcomere function, extracellular matrix remodeling, fibrosis, transcriptional regulation, oxidative stress, and cytoskeletal adaptation [34]. The same analysis also linked these molecular signatures to immune-cell infiltration, suggesting that structural remodeling and immune dysregulation may be biologically connected in HF [34].

Genetic and transcriptomic studies may help explain why patients with similar clinical risk factors develop different HF phenotypes. These approaches are not yet ready to replace clinical classification, and many candidate markers require further validation. However, they provide a foundation for future precision medicine by connecting inherited susceptibility and gene-expression changes with myocardial remodeling, immune activity, fibrosis, and cardiometabolic stress.

## 5. Immunologic Mechanisms in HFrEF and HFpEF

### 5.1 Inflammation as a Shared Driver of Heart Failure Progression

Inflammation is increasingly recognized as a common contributor to heart failure progression, although its origin differs across HF phenotypes. In HFrEF, inflammatory activation often follows direct myocardial injury. Ischemia, cardiomyocyte death, or toxic injury can initiate an immune response that is initially reparative but may become maladaptive as remodeling continues. In HFpEF, inflammation more often arises from systemic comorbidity burden. Conditions such as obesity, diabetes, aging, and chronic kidney disease can create a persistent low-grade inflammatory state that affects the coronary microvasculature and myocardium over time [35] (Figure 2).

Inflammatory signaling is closely linked to oxidative stress in both phenotypes. Reactive oxygen species can intensify proinflammatory pathways, while recruited immune cells can further increase oxidative injury within cardiac tissue [36,37]. Cytokines such as TNF-α, IL-1β, and IL-6 have been implicated in worsening ventricular function and adverse remodeling, supporting inflammation as an active contributor to HF biology rather than only a secondary marker of disease severity [37]. Clinical biomarker data further support this concept. In a biomarker sub study from the DAPA-HF study, higher VCAM-1 levels were associated with worse outcomes in HFrEF, suggesting that immune-cell adhesion and vascular inflammation may identify a clinically relevant inflammatory pathway [38].

Inflammation is therefore best understood as a shared remodeling pathway with phenotype-specific triggers rather than as a diagnostic separator between HFrEF and HFpEF.

### 5.2 Innate Immune Activation and Sterile Inflammation

Innate immune activation provides a mechanism by which noninfectious stressors contribute to heart failure progression. In HFpEF, systemic conditions such as obesity, diabetes, hypertension, and renal dysfunction can create a chronic inflammatory environment that affects the coronary microvasculature and myocardium [39]. Unlike infection-driven inflammation, sterile inflammation is triggered by endogenous stress signals released during metabolic dysfunction, oxidative injury, endothelial activation, and tissue damage.

Sterile inflammation can amplify myocardial remodeling through several interconnected mechanisms. Endothelial activation promotes leukocyte adhesion and trafficking into the myocardial and perivascular space, while tissue injury releases danger signals that activate innate immune pathways [39]. Inflammasome signaling provides one important link between cellular stress and inflammation. Neutrophil activation may also worsen microvascular injury by impairing capillary flow and contributing to local hypoxia, which can further reinforce inflammatory and fibrotic responses [39].

In HFpEF, sterile inflammation is best understood as a persistent remodeling environment rather than a transient response to injury. This process helps explain how extracardiac metabolic and vascular stress can become linked to myocardial inflammation and microvascular dysfunction.

### 5.3 Macrophages and Monocytes in Cardiac Remodeling

Macrophages and monocytes contribute to heart failure by linking myocardial stress to inflammation, repair, and fibrosis. Under normal conditions, resident cardiac macrophages help maintain tissue homeostasis and support adaptive responses to injury. However, during myocardial injury or chronic hemodynamic and metabolic stress, circulating monocytes are recruited into the heart and differentiate into macrophages that can amplify inflammation and remodeling [40-45]. The effect of these cells depends heavily on timing and context. Early macrophage activation may help clear cellular debris and initiate repair, but persistent activation can sustain cytokine signaling, fibroblast stimulation, and extracellular matrix accumulation.

In HFrEF, macrophage recruitment is often triggered by direct cardiomyocyte injury, particularly after ischemia or necrosis. Damage-associated signals attract inflammatory monocytes into the myocardium, where they participate in wound healing and scar formation [42,44,45]. When this response resolves appropriately, macrophages help stabilize injured tissue. When it remains active, however, the same repair program can contribute to adverse ventricular remodeling, progressive fibrosis, and worsening systolic dysfunction [40-45].

In HFpEF, macrophage activation is more often driven by chronic systemic stress rather than a single focal injury. Metabolic stress can push cardiac macrophages toward an inflammatory phenotype and promote signaling between macrophages and cardiomyocytes that affects hypertrophy, fibrosis, autophagy, and relaxation [46]. In hypertensive HFpEF, CXCR4-expressing monocytes and macrophages appear to promote myocardial inflammation and fibrosis through macrophage-derived CXCL3, which stimulates fibroblast activation and collagen deposition [47].

These phenotype-specific roles suggest that monocytes and macrophages should not be viewed simply as markers of inflammation. In HFrEF, they are closely tied to the transition from myocardial injury to scar formation and adverse ventricular remodeling. In HFpEF, they more often translate chronic metabolic and pressure-related stress into interstitial inflammation, fibrosis, and impaired diastolic reserve. This distinction is important because future immunomodulatory strategies will likely need to target specific immune-cell pathways within defined HF phenotypes rather than broadly suppress inflammation.

### 5.4 Cytokine Signaling and Fibroinflammatory Pathways

Cytokine signaling provides an important link between immune activation and fibrotic remodeling in heart failure. After myocardial injury or chronic stress, inflammatory cells and fibroblasts communicate through soluble mediators that help coordinate tissue repair. When this response resolves appropriately, inflammation supports clearance of damaged tissue and scar formation. However, persistent immune activation can shift this reparative process toward maladaptive fibrosis, with ongoing fibroblast activation, extracellular matrix deposition, myocardial stiffening, and progressive ventricular dysfunction [48].

Several cytokine pathways appear to contribute to this fibroinflammatory state. TGF-β remains one of the central profibrotic signals because it promotes fibroblast activation and myofibroblast differentiation. Inflammatory cytokines such as IL-1β, IL-6, and TNF-α can amplify endothelial dysfunction, oxidative stress, and immune-cell recruitment [48,49]. These pathways reinforce one another within the myocardial microenvironment, creating a cycle in which inflammation promotes fibrosis, and the fibrosis further sustains cellular stress.

This interaction may be particularly relevant in HFpEF, where systemic comorbidities create a chronic inflammatory background that affects the heart over time. Biomarkers associated with inflammation and remodeling, including soluble ST2 and GDF-15, have been linked to prognosis in chronic HF, supporting the clinical relevance of these pathways [49]. However, these markers do not always represent fibrosis alone. In HFpEF, GDF-15 appears to reflect broader physiologic stress, emphasizing that fibroinflammatory signaling often captures systemic disease activity as much as local myocardial remodeling [50].

Recent clinical data further support the presence of cytokine, chemokine, and inflammasome-related activity in HFrEF. In a cohort of patients with HF and LVEF below 50%, expression of inflammasome-associated genes, including NLRP-family receptors, caspases, Toll-like receptors, chemokine pathways, TGF-β, NF-κB, and related inflammatory mediators, varied according to nutritional and sarcopenia status (51). These findings support the measurability of inflammatory pathway activity in HF, although they do not establish whether these markers are causal drivers of remodeling or reflections of broader systemic disease activity.

IL-11 has also emerged as a potential mediator of cardiac fibrosis. Experimental data suggest that IL-11 signaling can promote fibroblast activation through ERK-dependent pathways, while IL-11 or IL-11 receptor inhibition reduces collagen expression, extracellular matrix remodeling, and cardiac fibrosis in preclinical models [52]. Chemokine signaling adds another layer to this process by regulating immune cell trafficking into the myocardium. For example, CCR5-related pathways illustrate how chemokine receptors can have context-dependent effects, helping coordinate immune repair in some settings while contributing to maladaptive inflammation, fibrosis, and HFpEF-like remodeling in others [53].

Clinical data further support the relevance of IL-6 signaling in HFrEF. In an exploratory analysis of DAPA-HF, elevated IL-6 and hs-CRP were common among patients with chronic ambulatory HFrEF, and nearly 40% had IL-6 concentrations above the assay-specific upper limit of normal [54]. Higher IL-6 levels were associated with worse symptoms, higher NT-proBNP and hs-troponin T, lower kidney function, and increased risk of worsening heart failure or cardiovascular death [54]. These findings suggest that IL-6 may help identify patients with greater inflammatory burden, although the study does not establish whether IL-6 is a causal driver of disease progression or a marker of more advanced heart failure [54].

Cytokine and chemokine signaling therefore link immune activation to structural remodeling in HF. Their effects depend strongly on disease context, with injury-associated repair pathways predominating in HFrEF and chronic cardiometabolic signaling predominating in HFpEF.

### 5.5 Immunologic Differences Between HFrEF and HFpEF

Although HFrEF and HFpEF share many inflammatory mediators, the organization of the immune response differs between phenotypes. In HFrEF, immune activation is often organized around myocardial injury, innate immune sensing, monocyte recruitment, macrophage-mediated repair, and fibroblast-driven scar formation. When repair does not resolve appropriately, this process can sustain extracellular matrix expansion and adverse ventricular remodeling [40,41,43-45,55-58]. In HFpEF, immune activation is less defined by a single injury-repair sequence and more by persistent low-grade inflammation across vascular, metabolic, and myocardial compartments. Comorbidity-related inflammation promotes endothelial activation, impaired nitric oxide signaling, immune-cell recruitment, and diffuse fibroinflammatory remodeling [33,36,46,47]. Thus, the key distinction is not that the two phenotypes use separate immune pathways, but that shared mediators produce different structural and functional consequences depending on the underlying disease context.

### 5.6 Therapeutic Implications of Targeting Inflammation

The growing recognition of immune activation in heart failure has created interest in therapies that target specific inflammatory and fibroinflammatory pathways. However, clinical studies suggest that inflammation is unlikely to be successfully treated as a broad, uniform target. Instead, emerging evidence supports a more selective approach in which specific pathways are matched to patients with measurable inflammatory activity.

Cytokine-directed therapy has provided some of the clearest evidence for this concept. IL-1 blockade with anakinra has been studied in HFpEF patients with elevated inflammatory markers and has shown reductions in C-reactive protein and NT-proBNP, suggesting biologic activity in an inflammation-enriched phenotype [39]. Canakinumab, which targets IL-1β, reduced cardiovascular events in post-myocardial infarction patients with elevated C-reactive protein, with secondary analyses suggesting possible relevance to heart failure outcomes [39]. These findings support IL-1 signaling as a modifiable pathway, although dedicated heart failure outcome data remain limited. IL-6 inhibition is also under investigation, with trials such as HERMES evaluating whether targeting systemic inflammation can reduce cardiovascular death, heart failure hospitalization, and urgent heart failure visits in HFpEF and HFmrEF [39].

Other strategies have focused on pathways that connect inflammation to oxidative stress and microvascular dysfunction. In the SATELLITE phase 2a trial, myeloperoxidase inhibition with AZD4831 produced strong target engagement and was well tolerated in patients with symptomatic heart failure and LVEF of at least 40%. However, the trial was stopped early, and improvement was not seen in secondary or exploratory measures such as coronary flow reserve, NT-proBNP, 6-minute walk distance, or echocardiographic parameters [59]. This suggests that inhibiting an inflammatory enzyme is feasible but also shows that biomarker modulation alone does not guarantee clinical benefit.

Biomarker-guided therapy may therefore be critical for future trial design. In a TOPCAT ancillary analysis, suPAR, a marker of innate immune activation, independently predicted worse outcomes in HFpEF, but spironolactone did not lower suPAR levels or show differential benefit based on baseline suPAR [60]. This finding suggests that inflammatory biomarkers may be most useful for identifying high-risk immune phenotypes, even when the tested therapy does not directly modify that pathway.

Cell-based immunomodulation has also been explored in HFrEF. In DREAM-HF, transendocardial mesenchymal precursor cell therapy did not meet its primary or secondary endpoints, but exploratory analyses showed improved LVEF and fewer myocardial infarction or stroke events, with stronger signals among patients with elevated hsCRP [61]. These results are not definitive, but they reinforce the idea that inflammation-enriched subgroups may respond differently to immunomodulatory therapies.

Several molecular targets are currently being studied. CXCR4/CXCL3 macrophage-to-fibroblast signaling and IL-11/ERK-mediated fibrotic remodeling are promising preclinical pathways because they directly connect immune-cell recruitment, fibroblast activation, and extracellular matrix deposition [47,52]. Mitochondrial and oxidative-stress–targeted agents may also indirectly reduce inflammatory injury. A recent meta-analysis found improvements in LVEF, NYHA class, mortality, and HF hospitalization with mitochondrial-targeted therapies, but the certainty of evidence was low due to heterogeneity, risk of bias, and limited HFpEF representation [62].

Targeted inflammation therapy in heart failure remains promising but investigational. The main lesson from current studies is that inflammatory pathways are biologically active and measurable, but clinical benefit likely depends on choosing the correct pathway, phenotype, and biomarker-enriched population. Future trials will likely need to move beyond broad anti-inflammatory treatment and instead test whether specific immune or fibroinflammatory mechanisms are driving disease progression in defined heart failure subgroups.

## 6. Current Treatment of HFrEF

Current treatment of HFrEF is centered on guideline-directed medical therapy (GDMT) that modifies disease progression rather than only improving symptoms. Treatment guidelines organize HFrEF pharmacotherapy around four foundational medication classes: RAAS inhibition, preferably with an ARNI when tolerated, evidence-based beta-blockers, mineralocorticoid receptor antagonists, and SGLT2 inhibitors [6]. This framework is supported by evidence showing that HFrEF progression can be modified by targeting pathways involved in ventricular dysfunction and remodeling. Although foundational therapies were established in earlier landmark trials, more recent studies have refined how GDMT is initiated and optimized in contemporary practice.

The core benefit of beta-blockers and mineralocorticoid receptor antagonists was established in earlier landmark trials. CIBIS-II, MERIT-HF, and the U.S. Carvedilol Heart Failure Study demonstrated improved survival with evidence-based beta-blockers, while RALES, EPHESUS, and EMPHASIS-HF established the benefit of mineralocorticoid receptor antagonism across severe HFrEF, post-myocardial infarction left ventricular dysfunction, and milder symptomatic systolic heart failure [63-68]. Mechanistic studies further support these benefits by showing that beta-blockade can promote reverse ventricular remodeling, while spironolactone may limit extracellular matrix turnover and myocardial fibrosis [68-71].

**Figure 3:**
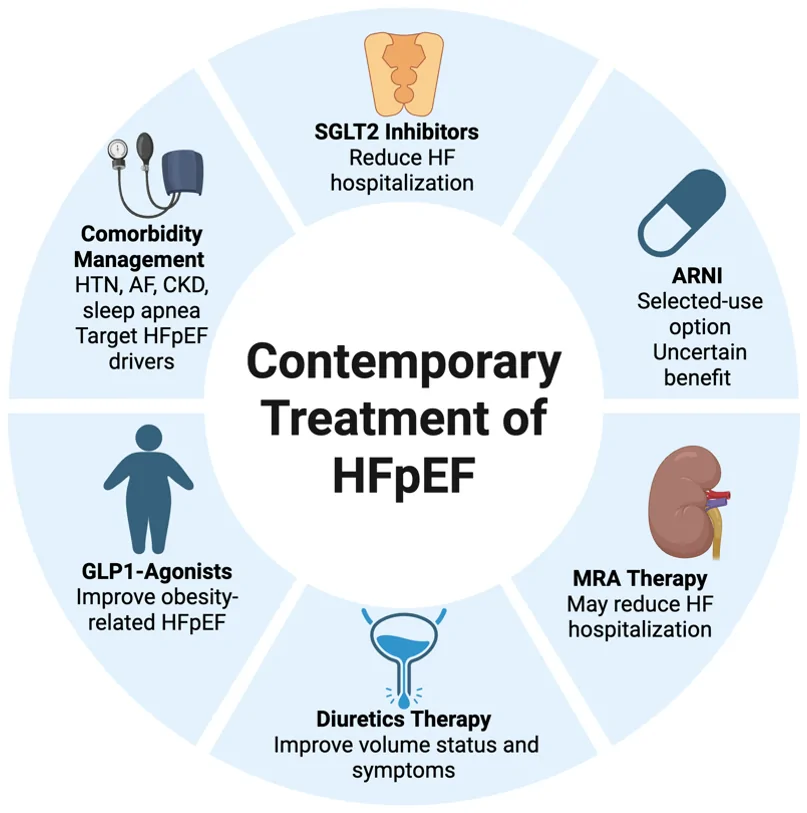
Contemporary treatment strategies in HFpEF. These include SGLT2 inhibitors, ARNI therapy selectively, mineralocorticoid receptor antagonism, decongestive therapy with diuretics, obesity-directed therapy, and comorbidity management.

More contemporary HFrEF therapy has expanded beyond traditional neurohormonal blockade (Figure 3). Angiotensin receptor-neprilysin inhibition improved outcomes beyond conventional RAAS inhibition and has become a central component of GDMT. In PARADIGM-HF, sacubitril/valsartan was superior to enalapril in reducing cardiovascular death or heart failure hospitalization, with additional reductions in all-cause mortality, cardiovascular mortality, and heart failure hospitalization [72]. PIONEER-HF also supported initiation of sacubitril/valsartan after stabilization from acute decompensated HFrEF produced a greater early reduction in NT-proBNP than enalapril without higher rates of major safety events [73]. Mechanistic data support this clinical benefit. In PROVE-HF, reductions in NT-proBNP after sacubitril/valsartan initiation correlated with improved LVEF and reduced LV volumes over 12 months, while EVALUATE-HF linked improvements in filling-pressure markers and left atrial remodeling with better patient-reported quality of life [74,75].

SGLT2 inhibitors further changed HFrEF management by demonstrating benefit regardless of diabetes status. EMPEROR-Reduced similarly showed that empagliflozin reduced cardiovascular death or heart failure hospitalization in patients with HFrEF, strengthening the evidence for SGLT2 inhibitors as a core HFrEF drug class [76]. In DAPA-HF, dapagliflozin reduced worsening heart failure or cardiovascular death in patients with symptomatic HFrEF receiving background recommended therapy, without increasing major adverse events related to volume depletion, renal dysfunction, or hypoglycemia [77]. Mechanistic trials suggest that these benefits may involve favorable cardiac remodeling rather than glucose lowering alone. EMPA-TROPISM showed improvement in LV volumes, LV mass, LVEF, exercise capacity, 6-minute walk distance, and quality of life in nondiabetic HFrEF, while SUGAR-DM-HF showed reduced LV end-systolic and end-diastolic volume indices and lower NT-proBNP in HFrEF with diabetes or prediabetes [78,79]. Dapagliflozin has also been associated with reduced epicardial adipose tissue and LV mass, suggesting potential cardiometabolic remodeling effects, although systemic inflammatory markers were not significantly changed [80].

Contemporary HFrEF care also emphasizes implementation, not just medication selection. STRONG-HF showed that early up-titration of GDMT with close follow-up after acute heart failure hospitalization reduced 180-day heart failure readmission or all-cause death compared with usual care [81]. This supports a shift away from slow, delayed medication adjustment and toward earlier achievement of target or maximally tolerated doses during high-risk periods.

Together, these studies reinforce GDMT as the foundation of HFrEF treatment while emphasizing that benefit depends not only on medication selection, but also on mechanism and timing. The evidence shows that HFrEF therapy can improve survival while also modifying the structural and physiologic changes that drive disease progression. However, many patients remain symptomatic or experience recurrent decompensation despite optimized GDMT, supporting the need for additional therapies in selected high-risk patients.

Vericiguat offers a complementary approach to HFrEF by targeting the nitric oxide–soluble guanylate cyclase–cyclic GMP pathway, which is involved in endothelial function, vascular tone, and myocardial stress. In the VICTORIA trial, vericiguat reduced the composite of cardiovascular death or first heart failure hospitalization in patients with chronic HFrEF and recent worsening heart failure, including those recently hospitalized or treated with intravenous diuretics [82]. This benefit was modest but clinically relevant because the trial focused on a high-risk population already receiving guideline-based therapy. In contrast, VICTOR studied more stable ambulatory patients with HFrEF and high background use of contemporary GDMT; in this lower-risk group, vericiguat did not significantly reduce the primary composite of first heart failure hospitalization or cardiovascular death [83]. Vericiguat may function as a targeted add-on therapy for selected patients with HFrEF and residual worsening risk, while its precise vascular and inflammatory mechanisms remain incompletely defined.

Ivabradine and hydralazine-isosorbide dinitrate provide additional evidence-based options for selected patients with persistent HFrEF risk despite foundational therapy. Ivabradine lowers heart rate through selective inhibition of the sinoatrial node funny current and may be useful in patients with symptomatic HFrEF who remain in sinus rhythm with an elevated resting heart rate despite maximally tolerated beta-blocker therapy. In SHIFT, ivabradine reduced the composite of cardiovascular death or heart failure hospitalization, with the benefit driven largely by fewer heart failure hospitalizations [84]. Hydralazine-isosorbide dinitrate targets vascular tone through complementary arterial and venous vasodilatory effects. It may be considered in patients who remain symptomatic despite GDMT, particularly in self-identified Black patients with advanced HFrEF. In A-HeFT, the addition of hydralazine-isosorbide dinitrate to background therapy improved clinical outcomes and reduced mortality and heart failure hospitalization in this population [85]. Together, these therapies illustrate that add-on treatment in HFrEF is most effective when matched to a specific physiologic or clinical phenotype rather than applied uniformly to all patients.

Other emerging therapies have explored more targeted approaches to residual HFrEF risk. Omecamtiv mecarbil, a cardiac myosin activator, was designed to improve systolic function by enhancing myosin cross-bridge formation and prolonging myocardial contraction. In GALACTIC-HF, it modestly reduced worsening heart failure events or cardiovascular death in patients with HFrEF and EF of 35% or less [86].

## 7. Current Treatment of HFpEF

Unlike HFrEF, treatment of HFpEF has historically been limited by the heterogeneity of the syndrome and the lack of therapies that consistently improve survival. Contemporary management therefore focuses on reducing heart failure events, relieving symptoms, and targeting the predominant pathophysiologic drivers, including congestion, myocardial fibrosis, impaired natriuresis, and cardiometabolic dysfunction.

Among currently available therapies, SGLT2 inhibitors have demonstrated the most consistent clinical benefit across the HFpEF spectrum. Although their precise mechanisms remain incompletely understood, they appear to reduce plasma volume and ventricular filling pressures, improve renal sodium handling, and favorably influence myocardial energetics and systemic metabolism. Consistent with these mechanisms, both EMPEROR-Preserved and DELIVER showed significant reductions in the composite of cardiovascular death or worsening heart failure, with the benefit driven primarily by fewer heart failure hospitalizations rather than a clear mortality reduction [87]. Mechanistic studies such as CAMEO-DAPA further demonstrated reductions in pulmonary capillary wedge pressure at rest and during exercise, supporting the hemodynamic effects observed in clinical practice [88].

Mineralocorticoid receptor antagonists represent another strategy aimed at limiting sodium retention, inflammation, and myocardial fibrosis. In TOPCAT, spironolactone did not significantly reduce the primary composite outcome, which consisted of cardiovascular death, aborted cardiac arrest, or heart failure hospitalization in the overall HFpEF population, but it was significantly associated with fewer heart failure hospitalizations [89]. More recently, FINEARTS-HF demonstrated that finerenone significantly reduced worsening heart failure events, providing stronger evidence that mineralocorticoid receptor antagonism may benefit patients with mildly reduced and preserved ejection fraction [90,91]. The recently published SOGALDI-PEF trial further showed that combining dapagliflozin with spironolactone produced greater reductions in NT-proBNP than dapagliflozin alone, although this was accompanied by greater potassium elevation and decline in renal function, highlighting the need to balance efficacy with safety when considering combination therapy [92].

Sacubitril/valsartan has produced more modest results in HFpEF. PARAGON-HF narrowly missed statistical significance for reducing cardiovascular death and total heart failure hospitalizations, while PARALLAX demonstrated greater reductions in NT-proBNP without corresponding improvements in exercise capacity or quality of life [93,94]. Similarly, PARAGLIDE-HF supported favorable biomarker effects after recent worsening heart failure but did not demonstrate a significant improvement in hierarchical clinical outcomes [95]. Collectively, these studies suggest that ARNI therapy may provide benefit in selected HFpEF phenotypes rather than uniformly across the entire population.

The emergence of obesity-targeted therapies further emphasizes the importance of phenotype-directed treatment in HFpEF. Semaglutide substantially improved symptoms, exercise capacity, body weight, and inflammatory markers in the STEP-HFpEF program, while SUMMIT demonstrated that tirzepatide reduced worsening heart failure events and improved health status in patients with obesity-related HFpEF [96,97]. These findings support the concept that correcting the metabolic abnormalities driving HFpEF can meaningfully improve both functional status and clinical outcomes.

Several additional therapies remain investigational or applicable only to selected patient populations. FAIR-HFpEF suggested that intravenous ferric carboxymaltose may improve exercise capacity in iron-deficient HFpEF patients, although the trial was underpowered because of early termination [98]. Likewise, accumulating evidence questions the routine use of beta-blockers in HFpEF. While they remain appropriate for comorbid conditions such as atrial fibrillation or ischemic heart disease, studies including PRESERVE-HR and more recent analyses have raised concern that beta-blockade may worsen chronotropic incompetence and exercise limitation in selected HFpEF patients, reinforcing the need for individualized rather than uniform treatment strategies [99,100].

Additional therapies in HFpEF remain largely supportive or investigational. Diuretics are still important for patients with congestion because they lower volume burden and filling pressures, but they should be viewed as symptom-directed therapy rather than proven disease-modifying treatment. In a TOPCAT subset analysis, greater loop diuretic use was associated with worse outcomes, likely reflecting a sicker and more congested HFpEF phenotype rather than demonstrating that diuretics themselves cause harm [101]. A post hoc analysis of PARAGON-HF similarly found that patients receiving higher baseline loop diuretic doses had a greater risk of subsequent HF events, further supporting loop diuretic requirement as a marker of congestion burden and disease severity [102]. Other therapies have been studied to target specific HFpEF mechanisms. Atorvastatin has shown short-term improvement in endothelial function and oxidative stress, suggesting possible vascular benefit, but outcome data are lacking [103]. Pirfenidone has been evaluated as an antifibrotic strategy, with PIROUETTE findings supporting myocardial fibrosis as a potential treatment target [104]. Colchicine has been studied as an anti-inflammatory approach and showed early signals of improved inflammatory markers and health status, although larger randomized trials are needed [105]. Mavacamten may be relevant for selected patients with hypercontractile or supranormal EF phenotypes by reducing excessive myosin-actin interaction and myocardial wall stress, but current HFpEF evidence remains preliminary [106]. Overall, these studies support a mechanism-based approach to HFpEF treatment, while emphasizing that none of these therapies currently has the broad outcome evidence established for SGLT2 inhibitors.

## 8. Knowledge Gaps and Future Directions

A central knowledge gap in heart failure is the limited translation of molecular phenotyping into clinically actionable classification. LVEF remains essential for diagnosis, prognosis, and treatment selection, but it does not fully reflect the biological heterogeneity within HF syndromes. This limitation is particularly relevant to HFpEF, where preserved systolic function can arise from overlapping pathways involving cardiometabolic inflammation, endothelial dysfunction, renal-metabolic stress, atrial and pulmonary vascular remodeling, and myocardial fibrosis. The relative contribution of these mechanisms likely differs across patients, which may partly explain the variable treatment responses observed in HFpEF trials.

Future progress in HFpEF may depend on identifying which molecular pathways are active, causal, and therapeutically modifiable. Mechanisms such as mitochondrial dysfunction, impaired nitric oxide signaling, titin-based stiffness, fibroblast activation, and immune-cell signaling are strongly implicated in HFpEF pathobiology. However, a biomarker or molecular abnormality may identify higher-risk biology without establishing that the pathway is causal or therapeutically targetable. Current biomarkers such as BNP and NT-proBNP remain clinically useful but do not fully capture the molecular diversity of HFpEF. More detailed molecular profiling approaches that examine proteins, metabolites, gene expression, and tissue-level patterns may help define biologically distinct subgroups. Longitudinal studies connecting molecular pathway activity with hemodynamic progression, myocardial remodeling, functional capacity, and clinical outcomes may help distinguish causal mechanisms from secondary markers of disease severity.

These considerations have important implications for HFpEF trial design. Broad enrollment based primarily on EF may attenuate therapeutic effects when only a subset of patients has the biological pathway targeted by the intervention. A more informative strategy may be to enrich trials using molecular, physiologic, or clinical features that align with the proposed mechanism of therapy. For example, antifibrotic therapies may be most relevant in patients with evidence of active fibrotic remodeling, whereas anti-inflammatory strategies may require identification of a defined inflammatory phenotype. Similarly, therapies targeting microvascular dysfunction, impaired energetics, or cardiometabolic stress may be better evaluated in patients with objective evidence of those abnormalities. This framework may help explain why several HFpEF therapies improve hospitalization risk, biomarkers, functional capacity, or selected subgroup outcomes without consistently demonstrating mortality benefit across broader trial populations.

A meaningful future direction is the integration of molecular biology with social determinants of health. HFpEF is closely linked to chronic cardiometabolic disease, and these risk factors are shaped by social and environmental exposures over time. Structural inequities, neighborhood environment, food access, insurance status, medication affordability, and access to preventive care can influence the development of hypertension, obesity, diabetes, and chronic kidney disease. These upstream conditions may also sustain downstream biological pathways such as systemic inflammation, endothelial dysfunction, and renal-metabolic stress. Therefore, molecular phenotyping alone is unlikely to fully explain HF risk or treatment response unless it is interpreted within the broader social context that shapes disease development and care delivery.

In HFrEF, the future challenge is different. The molecular basis of disease-modifying therapy is better established, and GDMT has substantially improved outcomes. Yet incomplete implementation continues to limit real-world benefit. Future advances may depend on improving the timing, sequencing, titration, adherence, affordability, and equitable delivery of established therapies, particularly after hospitalization. Additional agents may have the greatest value when applied to defined residual-risk profiles, such as recurrent worsening HF, persistent tachycardia, iron deficiency, vascular dysfunction, or ongoing remodeling despite optimized GDMT.

Future directions may therefore benefit from an integrated framework that evaluates HF through both molecular mechanism and clinical context. A useful precision approach must determine not only which pathway is abnormal, but whether it is causal, modifiable, and relevant to patient-centered outcomes. For HFpEF, integrating molecular phenotype with social determinants of health may help bridge the gap between mechanistic discovery and meaningful therapeutic progress.

## 9. Conclusion

Heart failure is a heterogeneous clinical syndrome shaped by interactions among myocardial injury, vascular dysfunction, metabolic stress, neurohormonal activation, and immune remodeling. Although LVEF remains central to clinical classification, it only partially captures the biological differences between HFrEF and HFpEF. HFrEF is more often characterized by cardiomyocyte injury, impaired systolic force generation, neurohormonal activation, and adverse ventricular remodeling, whereas HFpEF is more often driven by cardiometabolic stress, endothelial dysfunction, microvascular injury, inflammation, fibrosis, and impaired physiologic reserve.

These mechanistic differences help explain the divergent treatment landscapes of HFrEF and HFpEF. In HFrEF, shared pathways of neurohormonal activation and remodeling have enabled a standardized GDMT framework that improves survival and reduces hospitalization. In HFpEF, therapeutic progress has been limited by biological heterogeneity, suggesting that treatment benefit may depend on identifying the dominant clinical and molecular phenotype.

Future progress will require translating mechanistic insight into clinically meaningful treatment selection. Mechanism-based tools may improve classification and treatment selection, but their clinical value will depend on integration with comorbidity burden, access to care, and longitudinal treatment delivery. Integrating molecular biology with clinical and social context may allow HF care to become more precise, equitable, and responsive to the diverse pathways through which patients develop heart failure.

## Key points

• Heart failure is a heterogeneous clinical syndrome, and LVEF-based classification does not fully capture the biological diversity of HFrEF and HFpEF.

• HFrEF is primarily characterized by cardiomyocyte injury, impaired systolic contraction, neurohormonal activation, and adverse ventricular remodeling.

• HFpEF is driven by overlapping mechanisms including cardiometabolic stress, endothelial and microvascular dysfunction, inflammation, fibrosis, and impaired ventricular relaxation.

• Cellular and immunologic crosstalk among cardiomyocytes, mitochondria, endothelial cells, fibroblasts, macrophages, and cytokine pathways contributes to heart failure progression.

• Future heart failure care may benefit from integrating molecular phenotyping, clinical phenotype, comorbidity burden, and social determinants of health to improve treatment selection and equitable care.
